# Rap1GAP Mediates Angiotensin II-Induced Cardiomyocyte Hypertrophy by Inhibiting Autophagy and Increasing Oxidative Stress

**DOI:** 10.1155/2021/7848027

**Published:** 2021-04-15

**Authors:** Yan Gao, Di Zhao, Wen-zhi Xie, Tingting Meng, Chunxiao Xu, Yutong Liu, Pengfei Zhang, Xiuping Bi, Zhuo Zhao

**Affiliations:** ^1^Department of Cardiology, Jinan Central Hospital, Cheeloo College of Medicine, Shandong University, Jinan 250014, China; ^2^Department of Cardiology, Central Hospital Affiliated Shandong First Medical University, Jinan 250014, China; ^3^Department of Cardiology, The Third Affiliated Hospital of Shandong First Medical University (Affiliated Hospital of Shandong Academy of Medical Sciences), Jinan 250031, China

## Abstract

Abnormal autophagy and oxidative stress contribute to angiotensin II- (Ang II-) induced cardiac hypertrophy and heart failure. We previously showed that Ang II increased Rap1GAP gene expression in cardiomyocytes associated with hypertrophy and autophagy disorders. Using real-time PCR and Western blot, we found that Rap1GAP expression was increased in the heart of Sprague Dawley (SD) rats infused by Ang II compared with saline infusion and in Ang II vs. vehicle-treated rat neonatal cardiomyocytes. Overexpression of Rap1GAP in cultured cardiomyocytes exacerbated Ang II-induced cardiomyocyte hypertrophy, reactive oxygen species (ROS) generation, and cell apoptosis and inhibited autophagy. The increased oxidative stress caused by Rap1GAP overexpression was inhibited by the treatment of autophagy agonists. Knockdown of Rap1GAP by siRNA markedly attenuated Ang II-induced cardiomyocyte hypertrophy and oxidative stress and enhanced autophagy. The AMPK/AKT/mTOR signaling pathway was inhibited by overexpression of Rap1GAP and activated by the knockdown of Rap1GAP. These results show that Rap1GAP-mediated pathway might be a new mechanism of Ang II-induced cardiomyocyte hypertrophy, which could be a potential target for the future treatment of cardiac hypertrophy and heart failure.

## 1. Introduction

Cardiac hypertrophy is an adaptive response of the heart to various pathological stimuli, including pressure overload, myocardial infarction and ischemia, and hypoxia [1]. The heart is able to maintain normal functions in the hypertrophic compensatory stage by changing its structure and metabolism, but this compensatory mechanism causes an increased oxygen consumption, which eventually leads to ventricular dilatation and heart failure (HF) [2]. In clinical practice, cardiac hypertrophy is the main cause of cardiomyocyte death, decreased myocardial contractility, and electrophysiological disorders [3]. It is well established that renin-angiotensin system (RAS) plays a critical role in cardiac hypertrophy and heart failure. The main effector of RAS is angiotensin II (Ang II) [4], which results in ventricular remodeling by mechanisms including the regulating of cardiac autophagy and oxidative stress.

A growing body of evidence shows that the pathological process of cardiac hypertrophy is associated with excessive autophagy and reactive oxygen species (ROS), which eventually leads to cardiomyocyte necrosis and apoptosis [5, 6]. Autophagy is the process of using lysosomes to degrade the damaged organelles and macromolecules, which is essential for normal cell homeostasis. Cardiac autophagy plays an important role in maintaining cell activity and heart function under stresses. Autophagy-mediated clearance of damaged organelles reduces inflammasome activation, thus mitigating cardiomyocyte dysfunction and coronary microvascular injury [7]. The regulation and the related mechanisms of cardiac autophagy are unclear yet. It has been reported that ROS production in the heart might be involved in the autophagy regulation [8, 9]. Cardiac hypertrophy leads to increased oxygen consumption, and excessive ROS is produced in mitochondria, resulting in irreversible damage of mitochondrial DNA and further induces cardiac remodeling and failure [8, 9]. In the meanwhile, ROS regulates autophagy through various mechanisms involving catalase, Atg4, mitochondrial electron transport chains, and the Ca^2+^ release channel on the lysosomal membrane [10, 11].

The Ras superfamily protein is a kind of small molecule GTP-binding protein prevalent in eukaryotes, which is involved in many processes of cell activities, including cell proliferation and differentiation, membrane trafficking, cytoskeleton regulation, and intracellular oxidase formation. It has nine subfamilies including Ras and Rab subfamilies. The Rap protein acts as a molecular switch in the regulation of multiple signaling pathways [12]. It has five subtypes: Rap1a, Rap1b, Rap2c, Rap2b, and Rap2c. Among them, the most abundant subtypes in the heart are Rap1a and Rap1b. Studies have reported that Rap1 is associated with mitochondrial ROS production in the heart [13], while the inhibition of oxidative stress is considered a promising therapeutic strategy for pathological cardiac hypertrophy and heart failure. However, there are no studies regarding whether Rap1 is involved in the progression of cardiac remodeling. Rap1 GTPase-activating protein (Rap1GAP) converts active GTP-bound Rap1 to inactive GDP-bound state [14]. Our previous studies found that Rap1GAP is expressed in rat cardiomyocytes and is upregulated in Ang II-induced cardiomyocyte hypertrophy [15]. The present study using techniques of gene silence and overexpression further demonstrated that Rap1GAP plays a critical role in mediating Ang II-induced cardiomyocyte hypertrophy through its regulation on autophagy and oxidative stress.

## 2. Materials and Methods

### 2.1. Isolation of Neonatal Rat Cardiomyocytes and Cell Treatments

All animal procedures were approved by the Animal Care and Use Committee of Shandong University. Neonatal rat cardiomyocytes (NRCMs) were isolated enzymatically with collagenase II (Sigma-Aldrich, St. Louis, MO, USA) from 1- to 2-day-old Wistar rats. Briefly, the hearts of neonatal rats were cut into 1 mm^3^ pieces and digested with type 2 collagenase at 37°C for 5 min. The cardiomyocytes were counted and seeded in a 6-well culture plate after digestion, centrifugation, and purification. NRCMs were then cultured in Dulbecco's modified Eagle's medium (DMEM, Gibco, USA) supplemented with 8% horse serum (Gibco, USA), 5% fetal bovine serum (Gibco, USA), 1% penicillin/streptomycin (Hyclone, USA) and 0.1 mmol/l bromodeoxyuridine (Sigma-Aldrich, USA) for 72 h. Brdu was used to inhibit the proliferation of fibroblast.

Ang II (Cayman Chemical, Ann Arbor, MI, USA) was dissolved in saline and phenylephrine (Selleck Chemicals, Houston, TX, USA), 3-Methyladenine (Sigma-Aldrich, USA), and rapamycin (Sigma-Aldrich, USA) were dissolved in dimethyl sulfoxide (DMSO, Sigma-Aldrich). The following doses of reagents were used: 100 nM of Ang II, 10 *μ*M of phenylephrine, 10 mM of 3-Methyladenine, and 10 *μ*M of rapamycin. After 24 hours of treatment, cells were harvested for subsequent experiments.

### 2.2. Animal Study

All procedures were in compliance with the Guide for the Care and Use of Laboratory Animals and were approved by the Animal Care and Use Committee of Shandong University. Sprague Dawley (SD) adult rats at 8~10 weeks old were purchased from Beijing Vital River Laboratory Animal Technology Co., Ltd. Animals were fed with chow diet and maintained under a facility with a 12-hour light/dark cycle and constant temperature and humidity conditions. The animal model of cardiac hypertrophy was induced by subcutaneously injecting with Ang II (10 mg/kg) per day for 2 weeks [16–18], while rats were injected with an equal volume of saline solution as a control. Rats were individually euthanized and sacrificed with pentobarbital (150 mg/kg body weight, i.p.), and the left ventricles of the hearts were collected for further experiments.

### 2.3. Immunofluorescence Staining

NRCMs were plated on four-well glass chamber slides (Labtek, Germany) coated with 0.5% Gelatin (Gel) at a cell density of 60%. After 72 h, cells were washed in PBS for three times and then fixed in immunostaining fixture solution (Beyotime Biotechnology, Shanghai, China) for 10 minutes at room temperature, and nonspecific binding was blocked with goat serum blocking solution for 1 hour at room temperature. Cells were incubated with the Rap1GAP primary antibody (ab32373, Abcam, USA) or *α*-actinin (69758S, CST, USA) at 1 : 200 dilutions in goat serum blocking solution overnight at 4°C. The slides were washed three times with PBS and then incubated with Alexa Fluor 488 conjugated goat-anti-rabbit antibody (Invitrogen, USA) or Alexa Fluor 594 conjugated goat-anti-mouse antibody (Invitrogen, USA) for 30 minutes at room temperature. After washing three times with PBS, DAPI nuclei staining was performed. Cells were analyzed by a fluorescence microscope (Olympus Corporation, Tokyo, Japan), and the results of signals were quantified by the software of ImageJ.

### 2.4. Real-Time PCR

Total RNA from NRCMs was isolated using TRIzol reagent (Invitrogen, USA). cDNAs were synthesized by reverse transcription reagent kit according to the manufacturer's instructions (Toyobo, Japan). cDNA (0.5 *μ*g) was used for PCR amplification with gene-specific primers for Rap1GAP, atrial natriuretic factor (ANF), and brain natriuretic peptide (BNP). GAPDH expression was used for the quantitative internal control. The primers of related genes were as follows: Rap1GAP: forward 5′-GCTGAGTACGCCTGCTACAA-3′ and reverse 5′-CTTGTCATCGTCACCACCCA-3′; ANF: forward 5′-AGGGCTTCCTCCTG-3′ and reverse 5′-CCAGGTGGTCTAGCAGGTTC-3′; BNP: forward 5′-GCCAGTCTCCAGAACAATCC-3′ and reverse 5′-CCTTGGTCCTTTGAGAGCTG-3′; and GAPDH: forward 5′-CAGGGCTGCCTTCTCTTGTG-3′ and reverse 5′-GGTGATGGGTTTCCCGTTGA-3′. Relative gene expression levels to the control sample were calculated using the 2−*ΔΔ*Ct method. PCR was performed using LightCycler 480 (Roche, USA).

### 2.5. Western Blot Analysis

Total protein was isolated from the primary cardiomyocytes using RIPA buffer (Beyotime Biotechnology, Shanghai, China) with the protease inhibitor cocktail. Protein concentration was quantified with BCA protein assays kit (Beyotime Biotechnology, Shanghai, China). 30-70 *μ*g of proteins was separated by SDS-PAGE gel electrophoresis and then transferred to polyvinylidene fluoride membranes (Millipore, Bedford, MA, USA). The membranes were blocked by 5% nonfat dry milk with PBS containing 0.1% Tween-20 at room temperature for 1 h and then incubated with primary antibodies overnight at 4°C, followed by incubation with horseradish peroxidase- (HRP-) conjugated secondary antibodies at room temperature for 1 h. The primary antibodies used in this study were listed as follows: anti-Rap1GAP (1 : 10000, Abcam, Cambridge, UK), anti-LC3B I/II (1 : 1000; Cell Signaling Technology, MA, USA), anti-P62 (1 : 1000; Cell Signaling Technology), anti-Akt and anti-p-Akt (1 : 1000; Cell Signaling Technology), anti-mTOR and anti-p-mTOR (1 : 1000; Cell Signaling Technology), anti-P70s6k and anti-p-P70s6K (1 : 1000; Cell Signaling Technology), and GAPDH (1 : 1000; Cell Signaling Technology). Protein levels were normalized to GAPDH, and the results of signals were quantified by the software of ImageJ.

### 2.6. Rap1GAP Silencing by Small Interfering RNA

Lipofectamine RNAiMAX (Thermo Fisher, USA) was used for the transfection of small interfering RNA (siRNA) to silence Rap1GAP expression. Two target siRNAs against full-length Rap1GAP (si-Rap1GAP) were designed and synthesized by GenePharma (Shanghai GenePharma Co. Ltd., Shanghai, China) (no. 1, 715-735, 5′-CTGGTCTTCTCGCTCAAGTAT-3′; no. 2, 1004-1024, 5′-TGTCCACCAAGCTGCCATACA-3′). NRCMs were seeded in six-well plates (6 × 10^5^ per well). Lipofectamine RNAiMAX reagent was diluted, and siRNA in Opti-MEM medium was mixed following manufacturer's instructions. Cells were transfected with Rap1GAP siRNA (20 *μ*M) using 7.5 *μ*l of Lipofectamine RNAiMAX in 2 ml of DMEM containing 8% horse serum and 5% fetal bovine serum. 48 hours after the transfection, the medium was replaced with fresh culture medium and treated with 100 nM of Ang II for 24 hours. The knockdown efficiency of siRNA against Rap1GAP was determined by RT-PCR and Western blot. Scramble-siRNA (si-control) was used as negative control.

### 2.7. Ad-Rap1GAP Adenovirus Infection

The recombinant adenovirus vectors that overexpress Rap1GAP (Ad-Rap1GAP) adenovirus vectors were designed and synthesized by Vigene Bioscience, Inc. (Jinan, China). The virus titer was detected by the method of gradient dilution, and the final measured Rap1GAP adenovirus titer was 4.8 × 10^10^ pfu/ml. The adenovirus vectors were added to the NRCMs at a multiplicity of infection of 10 when the cell density reaches about 80% confluence. At 24 h after infection, cells were used for subsequent experiments.

### 2.8. Autophagic Flux Measurements by mRFP-GFP-LC3 Adenovirus Infection

Intracellular autophagic flux was quantified by the method of mRFP-GFP-LC3 adenoviral vector infection. The recombinant adenovirus vectors that mRFP-GFP-LC3 adenovirus vectors were designed and synthesized by Vigene Bioscience, Inc. (Jinan, China). NRCMs were infected with mRFP-GFP-LC3 adenovirus vectors at 50 MOI and incubated for 48 h before experiments. The cells were washed three times with PBS and then fixed with 4% paraformaldehyde at room temperature. Cells were viewed by a fluorescence microscope (Olympus Corporation, Tokyo, Japan). Autophagy flux was evaluated by counting of the cells with green puncta, red puncta, and yellow puncta. At least 50 cells were scored per sample in each experiment.

### 2.9. Transmission Electron Microscopy (TEM)

When cells cultured in 10 cm petri dishes (Corning; NY, USA) reached 80–90% confluence, cell were collected, centrifuged, washed with PBS, and immediately fixed in 2.5% glutaraldehyde fixative solution for 2 h at room temperature. The cells were then further fixed with a 1% citric acid solution for 1.5 h in the dark followed by dehydration with series concentrations (50%, 70%, 80%, and 90%) of ethanol and embed in the resin overnight. Ultrathin sections of 70 nm thickness were cut with an ultramicrotome (UC7, Leica, Germany) and collected on 200-mesh copper grids. The sections were stained with 2% aqueous solution of uranyl acetate for 20 min in the dark and incubated in lead citrate solution for 7 min. Autophagosomes were examined with an electron microscope (Hitachi HT-7800) operating at 80 kV.

### 2.10. Reactive Oxygen Species (ROS) Measurement

Intracellular ROS production in cardiomyocytes was assessed by the fluorescence intensity of dihydroethidium (DHE, Beyotime Biotechnology) staining. After the cardiomyocytes were subjected to their respective treatments, they were incubated with 10 *μ*M DHE at 37°C for 30 min. The cells were washed with PBS for 3 times, and then, the adherent cells were immediately observed under a fluorescence microscope (Olympus Corporation, Tokyo, Japan). ImageJ software was used to quantify the fluorescence intensity of each picture.

### 2.11. Hematoxylin and Eosin (HE) Staining

The tissues of the left ventricle were fixed in 4% formalin and embedded in paraffin and then cut into 5 *μ*m serial sections. Tissue sections were floated onto a warm water bath from where they were placed on slides. After deparaffinizing in xylene and rehydrating in graded ethanol solutions, the sections were stained with eosin solution. Sections were then examined using a microscope (Olympus Corporation, Tokyo, Japan), and the cell surface areas were measured using Image-Pro Plus 6.0 software. A random collection of 10 cardiomyocyte images is calculated, which contains at least 25 cells from the cross-sectional area of cardiomyocytes.

### 2.12. Echocardiography

Rats were lightly anaesthetized with 1.5%-2% isoflurane via inhalation. Anaesthetized rats were subjected to a transthoracic echocardiography, using a Vevo 770 ultrasound with a 25 MHz transducer (Visual Sonics, Toronto, Canada). The left ventricular ejection fraction (EF), fractional shortening (FS), left ventricular end-systolic dimension (LVESD), and left ventricular end-diastolic dimension (LVEDD) were calculated from the M-mode recording. All parameters were collected from at least three consecutive cardiac cycle measurements and averaged.

### 2.13. TUNEL Staining

Apoptosis was determined by terminal deoxyribonucleotidyl transferase UTP nick end labelling (TUNEL) kit (Roche, Switzerland) according to the manufacturer's instructions. The percentage of apoptotic nuclei per section was calculated by counting the number of TUNEL-positive cardiomyocyte nuclei divided by the total number of DAPI-positive nuclei. Image-Pro Plus software (Image Solutions, Torrance, CA, USA) was used to count the cells and calculate the average value. Apoptotic cell number was counted in at least five randomized microscope fields in each of three independent samples under a fluorescence microscope.

### 2.14. Flow Cytometry for Cell Apoptosis Assay

The Annexin V-PE/7AAD apoptosis detection kit (KeyGEN BioTECH Co., Nanjing, China) was used to examine the cell apoptosis in different groups. Briefly, NRCMs were incubated in six-well plates at 6 × 10^5^ cells/well. After different treatments, NRCMs were collected and resuspended in binding buffer, flowed by incubation with Annexin V-phycoerythrin (PE) and 7-aminoactinomycin D (7AAD) at room temperature for 15 min. C6 Flow Cytometer™ system (BD Biosciences, CA, USA) was used to analyze the apoptotic rate of cells.

### 2.15. Statistical Analysis

Statistical analysis was performed using GraphPad Version 8.0 (GraphPad Software, La Jolla, CA, USA). All data were expressed as the mean ± SEM. Student's *t*-tests were performed to compare means between two groups. ANOVA followed by Bonferroni's multiple comparisons was used to compare means from three or more groups. A level of *P* < 0.05 was considered to be statistically significant. All experiments were performed independently three times.

## 3. Results

### 3.1. Ang II Induced Cardiomyocyte Hypertrophy, Oxidative Stress, and Apoptosis and Inhibited Autophagy

**Figure 1:**
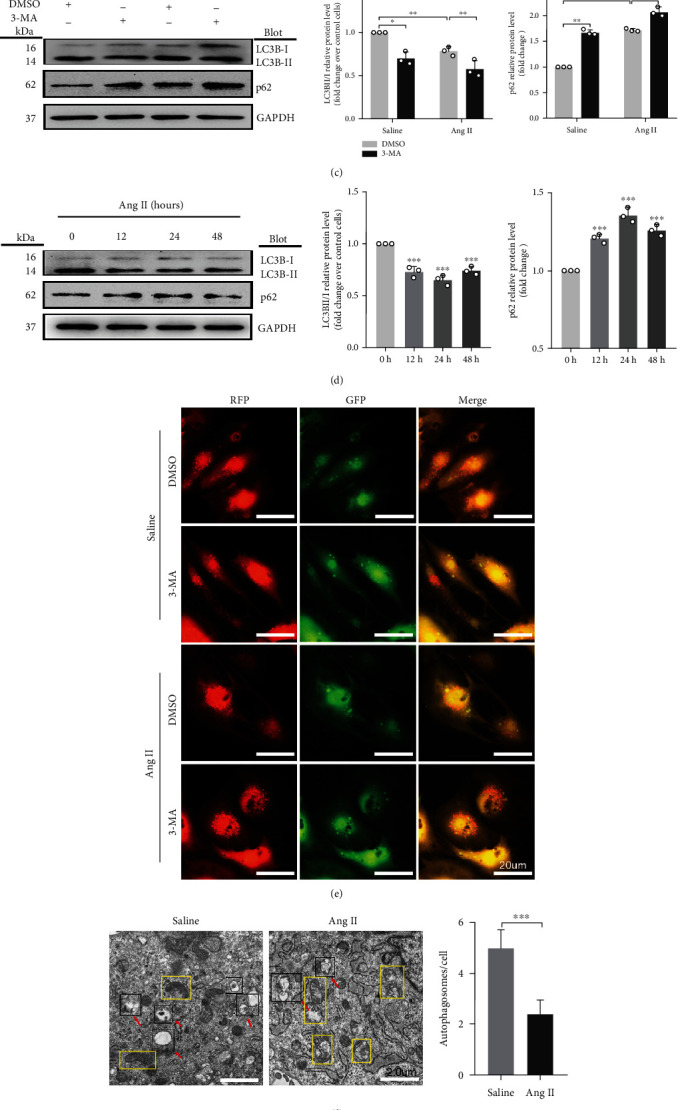
Ang II induces hypertrophy, oxidative stress, and apoptosis and inhibits autophagy in cardiomyocytes. (a) Cardiomyocyte surface area determined by immunofluorescence staining. Scale bar: 50 *μ*m, *n* = 25. (b) Relative mRNA levels of ANF and BNP in Ang II- and vehicle-treated cardiomyocytes, determined using qRT-PCR. (c) Western blot analysis for the expression of LC3B and p62 in cardiomyocytes treated with 3-MA. (d) Western blot analysis for the expression of LC3BII/I and p62 in cardiomyocytes treated with Ang II at different time points. (e) NRCMs infected with adenovirus mRFP-GFP-LC3 and analyzed using a fluorescence microscope. Scale bar: 20 *μ*m. (f) Autophagy activity assessed using TEM, with red arrows indicating autophagy lysosome or autophagosome and the yellow boxes indicating mitochondria. Scale bar: 2.0 *μ*m. (g) ROS production in cardiomyocytes quantified by DHE fluorescent dyes. Scale bar: 50 *μ*m. (h) Apoptosis levels measured by TUNEL staining. Red: apoptotic cells; blue: nuclei DAPI staining. Scale bar: 100 *μ*m. ^∗^*P* < 0.05 and ^∗∗^*P* < 0.01. All results are the mean ± SEM of three independent experiments (*n* = 3).

Compared with vehicle-treated cells, Ang II significantly increased cell surface area (*P* < 0.01; Figure 1(a)) and the mRNA expression of ANF and BNP (*P* < 0.05; Figure 1(b)), suggesting that Ang II induced hypertrophy in the NRCMs.

To determine the effect of Ang II on autophagy, we measured the markers of autophagy, LC3BII/I and p62 expressions, in the absence or presence of autophagy inhibitor 3-Methyladenine (3-MA). Compared with vehicle-treated cells, 3-MA reduced the ratio of LC3BII/I and increased the content of p62 (*P* < 0.05 and *P* < 0.01, respectively; Figure 1(c)). Consistent with the results of 3-MA, Ang II also inhibited autophagy (*P* < 0.01; Figure 1(c)). Subsequently, we measured the markers of autophagy at series time points of Ang II stimulation. Compared with 0 h control, the ratio of LC3BII/I expression reached the lowest level at 24 hours and then gradually increased but still lower at 48 hours than that at 0 h (*P* < 0.01; Figure 1(d)). The level of p62 significantly increased after Ang II treatment and reached the highest level at 24 hours (*P* < 0.01; Figure 1(d)). The changes of cardiomyocyte autophagy by Ang II were further confirmed by the experiment of mRFP-GFP-LC3 adenovirus infection in cardiomyocytes. As shown in Figure 1(e), the autophagic flux after Ang II or 3-MA treatment was decreased by reducing the autophagosome conversion to autophagolysosome, as indicated by increase of yellow dots (indicating autophagosome) and decrease of free red dots (indicating autophagolysosome). Compared to those treated with vehicle, autophagosomes and autophagic lysosomes scanned by TEM were significantly decreased (indicated by the red arrow) (*P* < 0.01; Figure 1(f)), and mitochondrial disruption was observed in the yellow box in cardiomyocytes treated with Ang II. All these data suggest that autophagy was markedly decreased by Ang II treatment.

To assess the mechanism of Ang II-induced cardiomyocytes hypertrophy, we analyzed the intracellular ROS and cell apoptosis in cardiomyocytes treated with Ang II. ROS production measured by dihydroethidium (DHE) staining was markedly increased in Ang II-treated NRCMs in comparison to vehicle-treated cells (*P* < 0.01; Figure 1(g)). TUNEL staining showed that Ang II increased cardiomyocyte apoptosis (Figure 1(h)).

### 3.2. Rap1GAP Is Upregulated in Ang II-Induced Cardiomyocyte Hypertrophy

**Figure 2:**
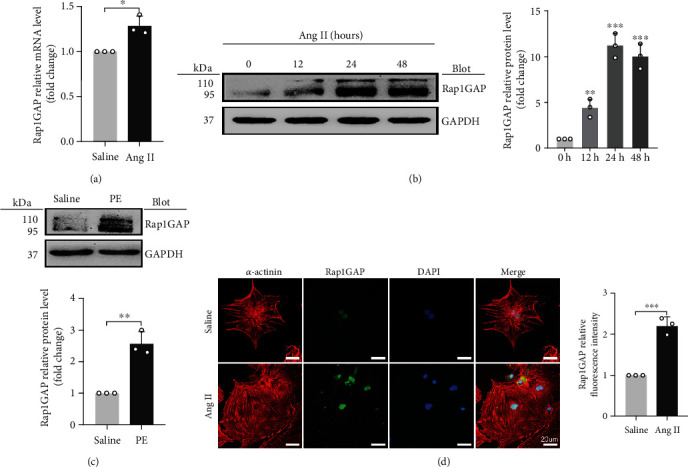
Rap1GAP is upregulated in hypertrophic cardiomyocytes. (a) mRNA level of Rap1GAP in vehicle- or Ang II-treated cardiomyocytes determined using qRT-PCR. (b) Western blot analysis for Rap1GAP in Ang II-treated cardiomyocytes at different time points. (c) Western blot analysis for Rap1GAP expression in phenylephrine-treated cardiomyocytes. (d) The expression level and localization of Rap1GAP detected by double-label immunofluorescence staining. Scale bar: 20 *μ*m. ^∗^*P* < 0.05 and ^∗∗^*P* < 0.01. All results are the mean ± SEM of three independent experiments (*n* = 3).

Consistent with our previous results from tandem mass tag (TMT) protein mass spectrometry and bioinformatics analysis [15], Rap1GAP mRNA levels were significantly increased in Ang II-induced hypertrophic cardiomyocytes (*P* < 0.05; Figure 2(a)). Western blot analysis further demonstrated that Rap1GAP protein expression was also increased by Ang II at different time points. As shown in Figure 2(b), the peak increase of Rap1GAP protein expression was at 24 h of Ang II treatment, which was gradually decreased but still remained higher at 48 h than that at 0 h (*P* < 0.01). In addition, we also found that the protein expression of Rap1GAP was increased in phenylephrine-treated cardiomyocytes (*P* < 0.01; Figure 2(c)).

Immunofluorescence staining showed that Rap1GAP was expressed in both the cytoplasm and nuclei, but mainly in the nucleus. Compared with the vehicle-treated cells, the level of Rap1GAP in NRCMs treated with Ang II was markedly elevated (*P* < 0.01; Figure 2(d)).

### 3.3. Cardiac Rap1GAP Is Increased in Sprague Dawley Rats Infused by Ang II

**Figure 3:**
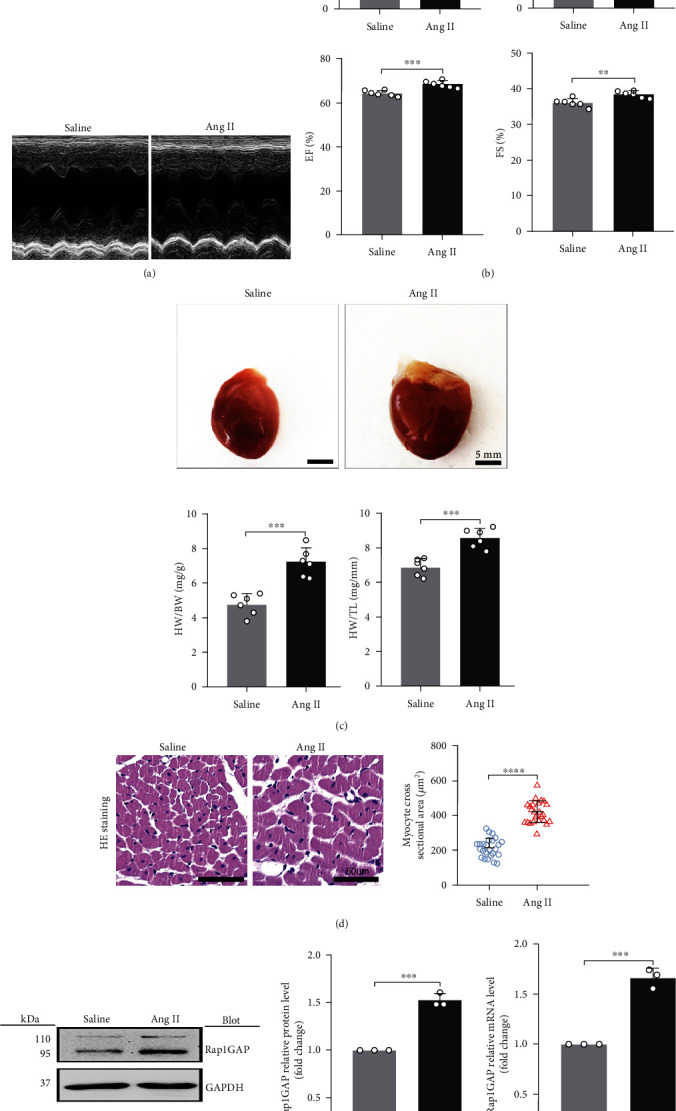
Cardiac Rap1GAP is upregulated in Sprague Dawley rats infused by Ang II. (a) Representative M-mode echocardiographic images. (b) Statistical results of the LVEDD, LVESD, EF, and FS at 2 weeks after Ang II or saline infusion; *n* = 6. (c) Representative gross heart pictures (scale bar: 5 mm) and HW/BW and HW/TL ratios; *n* = 6. (d) Representative images of HE staining in left ventricle transverse sections (scale bar: 50 *μ*m, *n* = 25). (e) Western blot analysis for Rap1GAP in the hearts of saline-treated rats and Ang II-treated rats. (f) mRNA level of cardiac Rap1GAP in saline-treated rats and Ang II-treated rats. ^∗^*P* < 0.05 and ^∗∗^*P* < 0.01. All results are the mean ± SEM of three independent experiments (*n* = 3).

To further investigate if our findings from in vitro studies are consistent with in vivo study, animal model of cardiac hypertrophy was induced by chronic treatment of Ang II for 2 weeks [16–18]. Echocardiographic results showed that the left ventricular end-diastolic diameter (LVEDD) and left ventricular end-systolic dimension (LVESD) were remarkably decreased (*P* < 0.01; Figures 3(a) and 3(b)), while the ejection fraction (EF) and fractional shortening (FS) were increased in Ang II-treated rats (*P* < 0.01; Figures 3(a) and 3(b)). Ang II induced cardiac hypertrophy, as reflected by the increased ratios of heart weight (HW)/body weight (BW) and HW/tibia length (TL) (*P* < 0.01; Figure 3(c)). The Ang II-induced cardiac hypertrophy was also confirmed by the hematoxylin and eosin (HE) staining in left ventricles, indicating that the cardiomyocytes in Ang II-treated rats were significantly larger than those in saline-treated rats (*P* < 0.01; Figure 3(d)).

We measured the cardiac Rap1GAP protein and mRNA levels in the cardiac hypertrophy model compared to the control rats. The results showed that both protein and mRNA levels of Rap1GAP in the heart were higher in Ang II versus saline-treated rats (*P* < 0.01; Figures 3(e) and 3(f)).

### 3.4. Rap1GAP Knockdown Increases Autophagy and Attenuates Oxidative Stress in Ang II-Treated Cardiomyocytes

**Figure 4:**
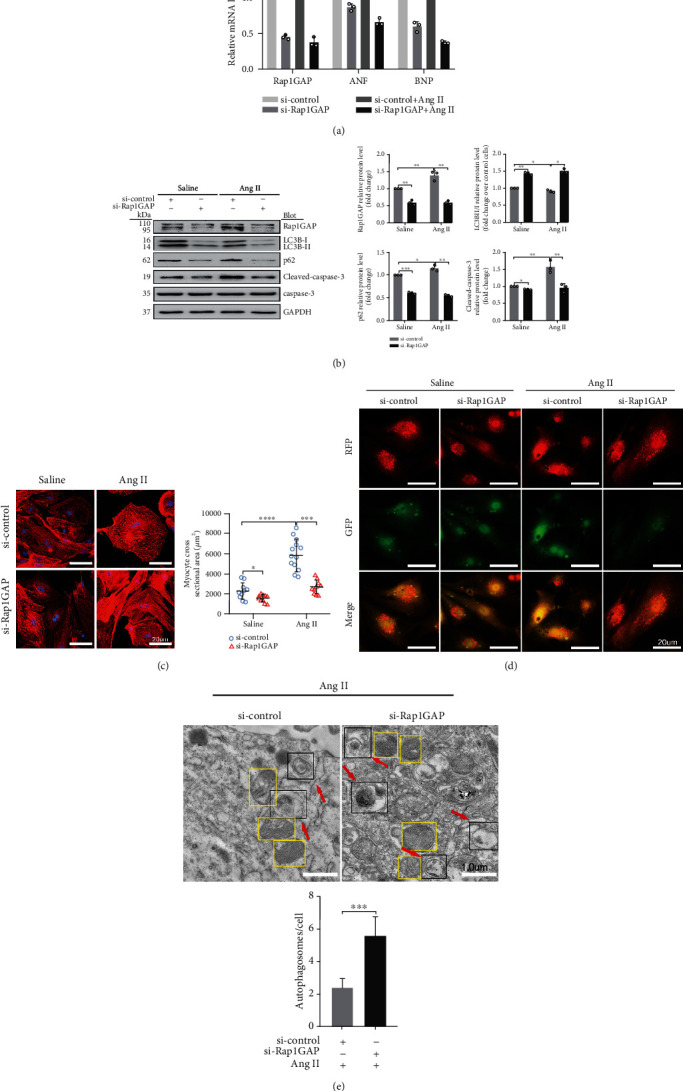
Rap1GAP knockdown attenuates cardiomyocyte hypertrophy and enhanced Ang II-induced autophagy. (a) mRNA levels of Rap1GAP, ANF, and BNP in Rap1GAP knockdown and intact cardiomyocytes determined using RT-PCR. (b) Western blot analysis for LC3BII/I, p62, and cleaved-caspase-3/caspase-3 in Rap1GAP-deficient cardiomyocytes. (c) Representative images and the quantifications from *α*-actinin staining showing cardiomyocyte surface area in Rap1GAP-deficient cardiomyocytes. Scale bar: 20 *μ*m; *n* = 10. (d) Representative images from a fluorescence microscope showing Rap1GAP knockdown vs. intact NRCMs infected with adenovirus mRFP-GFP-LC3. Scale bar: 20 *μ*m. (e) Representative images and quantifications from TEM showing autophagy lysosomes, autophagosomes, and mitochondria. The red arrows indicating autophagy lysosome or autophagosome and the yellow boxes indicating mitochondria. Scale bar: 1.0 *μ*m. ^∗^*P* < 0.05 and ^∗∗^*P* < 0.01. Results are the mean ± SEM of three independent experiments (*n* = 3).

To further determine the functional role of Rap1GAP in Ang II-induced cardiomyocyte hypertrophy, neonatal rat cardiomyocytes were transfected with small interfering RNA against Rap1GAP (si-Rap1GAP) or scrambled control (si-control), followed by Ang II stimulation for 24 hours. RT-PCR indicated that compared with the si-control group, the Rap1GAP mRNA was significantly reduced by approximately 60% after si-Rap1GAP transfection (*P* < 0.05, Figure 4(a)). Consistently, Rap1GAP siRNA reduced the protein expression of Rap1GAP compared to control siRNA (*P* < 0.01; Figure 4(b)). Moreover, knockdown of Rap1GAP dramatically decreased Ang II-induced expression of ANF and BNP compared with the si-control (*P* < 0.05 or 0.01; Figure 4(a)). Meanwhile, immunostaining of NRCMs for *α*-actinin showed that knockdown of Rap1GAP markedly attenuated the increase in cardiomyocyte hypertrophy induced by Ang II (*P* < 0.01; Figure 4(c)).

Next, we evaluated the effects of si-Rap1GAP on autophagy in cardiomyocytes. As shown in Figure 4(b) in both vehicle- and Ang II-treated cells, compared with si-control, the expression of LC3BII/I was increased and the level of p62 was decreased after Rap1GAP knockdown (*P* < 0.05 or 0.01). In the experiment of mRFP-GFP-LC3 adenovirus infection, compared with the si-control group, Rap1GAP knockdown increased the red dots and decreased the merged yellow spots, and the binding of autophagosomes to lysosomes was blocked. The data indicated that Rap1GAP deficiency enhanced the Ang II-induced autophagy flux (Figure 4(d)). TEM results also revealed that si-Rap1GAP mitigated Ang II-induced reduction of autophagosomes and autolysosomes in cardiomyocytes and improved Ang II-induced mitochondrial fractures (*P* < 0.01; Figure 4(e)).

**Figure 5:**
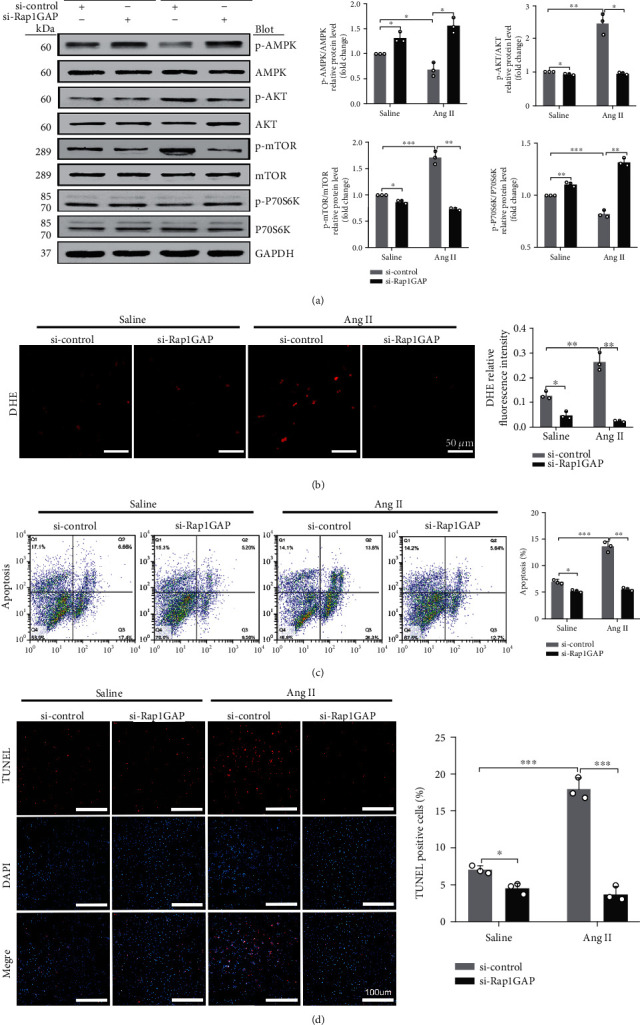
Rap1GAP knockdown inhibited Ang II-induced ROS production and apoptosis. (a) Phosphorylated protein and total protein expression of AMPK, AKT, mTOR, and p70s6K in Rap1GAP knockdown vs. control cardiomyocytes. (b) ROS production in Rap1GAP knockdown and intact cardiomyocytes evaluated using DHE fluorescent dyes. Scale bar: 50 *μ*m. (c) Flow cytometric analysis for NRCM apoptosis stained with 7AAD and Annexin V-PE. In the coordinate system, *X* axis is 7AAD, and *Y* axis is Annexin V-PE. (d) TUNEL staining for cardiomyocyte apoptosis assay. Scale bar: 100 *μ*m. ^∗^*P* < 0.05 and ^∗∗^*P* < 0.01. Data are the mean ± SEM of three independent experiments (*n* = 3).

To further study the mechanisms and the involved signaling pathways by which Rap1GAP regulates autophagy, we examined the activity of the AMPK/AKT/mTOR pathway which was the classical pathway of autophagy regulation. The results showed that Rap1GAP knockdown significantly decreased the expression of p-AKT and p-mTOR (*P* < 0.05 and *P* < 0.01, respectively) and increased the expression of p-AMPK and p-p70s6k (*P* < 0.05 and *P* < 0.01, respectively; Figure 5(a)).

We also examined the effects of Rap1GAP knockdown on oxidative stress and apoptosis in Ang II-induced hypertrophic cardiomyocytes. Compared with control siRNA, Rap1GAP knockdown reduced the ROS generation in both vehicle- and Ang II-treated cardiomyocytes (*P* < 0.01; Figure 5(b)). The ratio of cleaved-caspase-3/caspase-3 was reduced in Rap1GAP-deficient cardiomyocytes compared to Rap1GAP intact cells (*P* < 0.01; Figure 4(b)). Similarly, knockdown of Rap1GAP significantly inhibited Ang II-induced cardiomyocyte apoptosis, demonstrated by TUNEL and Annexin V PE/7AAD staining (*P* < 0.01; Figures 5(c) and 5(d)).

### 3.5. Rap1GAP Overexpression Reduced Autophagy and Increased Oxidative Stress in Ang II-Treated Cardiomyocytes

**Figure 6:**
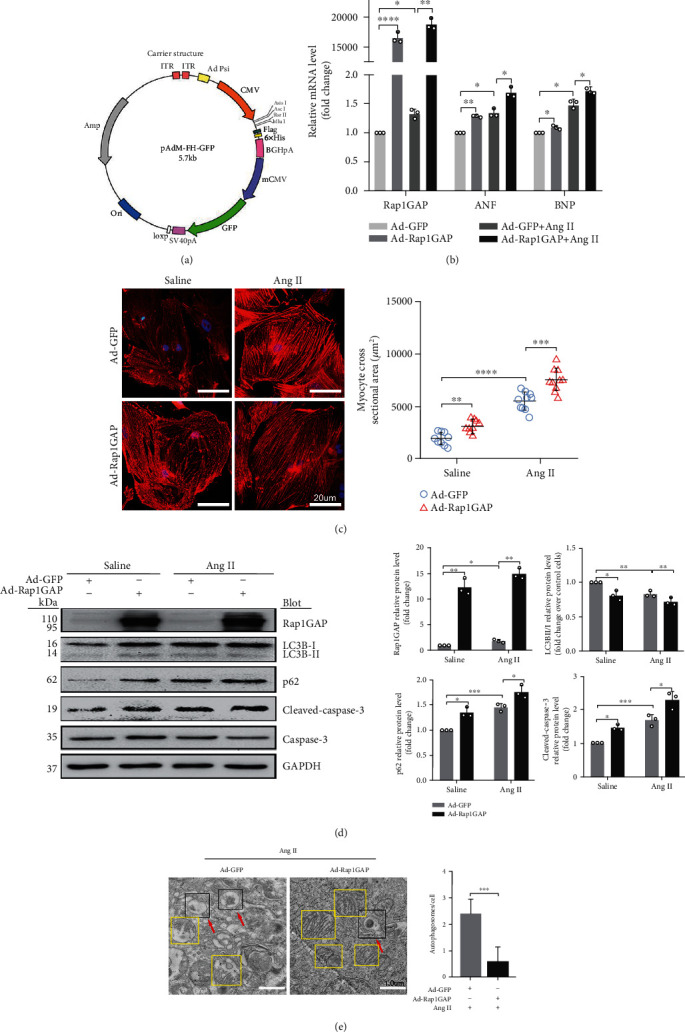
Rap1GAP overexpression aggravates cardiomyocyte hypertrophy and further inhibits Ang II-induced autophagy. (a) The structure of the construct of expression plasmid. (b) mRNA levels of Rap1GAP, ANF, and BNP in Rap1GAP overexpressing and intact cardiomyocytes determined using RT-PCR. (c) Representative images and quantification from *α*-actinin staining showing cardiomyocyte surface area in Rap1GAP overexpressing and intact cardiomyocytes. Scale bar: 20 *μ*m; *n* = 10. (d) Western blot analysis for LC3BII/I, p62, and cleaved-caspase-3/caspase-3 in Rap1GAP overexpressing and intact cardiomyocytes. (e) Representative images and quantifications from TEM showing autophagy lysosomes, autophagosomes, and mitochondria. The red arrows indicate autophagy lysosome or autophagosome, and the yellow boxes indicate mitochondria. Scale bar: 1.0 *μ*m. ^∗^*P* < 0.05 and ^∗∗^*P* < 0.01. Results are the mean ± SEM of three independent experiments (*n* = 3).

Figure 6(a) shows the structure of the Rap1GAP plasmid construct. The gene transduction rate of the viral vector measured by flow cytometry analysis was 85.4%, which was consistent with the result of GFP immunofluorescence image (Figure S1). Compared to control vector (Ad-GFP), the cardiomyocytes infected with adenovirus vector overexpressing Rap1GAP (Ad-Rap1GAP) increased the content of Rap1GAP (*P* < 0.01; Figure 6(b)) and enhanced the expression of ANF and BNP (*P* < 0.05; Figure 6(b)) after Ang II treatment. Moreover, Rap1GAP overexpression dramatically increased the hypertrophic growth of cardiomyocytes in response to Ang II (*P* < 0.01; Figure 6(c)). Rap1GAP overexpression dramatically decreased the level of autophagy in Ang II-treated cardiomyocytes, demonstrated by the decreased ratio of LC3BII/I and the increased p62 (*P* < 0.05 or *P* < 0.01; Figure 6(d)). Consistently, TEM results showed that overexpression of Rap1GAP aggravated Ang II-induced decrease of autophagosomes and increase of mitochondrial fractures compared with the Ad-GFP group (*P* < 0.01; Figure 6(e)).

**Figure 7:**
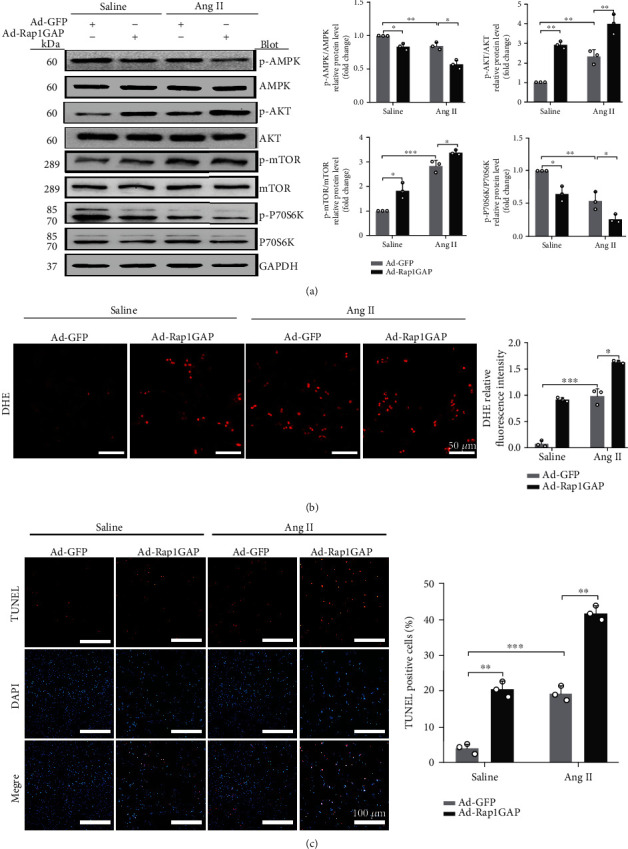
Rap1GAP overexpression aggravates Ang II-induced ROS production and apoptosis. (a) Phosphorylated protein and total protein expression of AMPK, AKT, mTOR, and p70s6K measured using Western blot analysis. (b) ROS production in Rap1GAP overexpressing and control cardiomyocytes evaluated using DHE fluorescent dyes. Scale bar: 50 *μ*m. (c) Cell apoptosis determined by TUNEL staining. Scale bar: 100 *μ*m. ^∗^*P* < 0.05 and ^∗∗^*P* < 0.01. Data are the mean ± SEM of three independent experiments (*n* = 3).

In contrast to Rap1GAP knockdown, Rap1GAP overexpression increased the expression of p-AKT and p-mTOR (*P* < 0.01 and *P* < 0.05, respectively) and reduced the expression levels of p-AMPK and p-p70s6k compared with Ad-GFP control (*P* < 0.05; Figure 7(a)).

Moreover, the overexpression of Rap1GAP increased oxidative stress and promoted apoptosis in cardiomyocytes. DHE staining showed that Ad-Rap1GAP significantly increased the generation of ROS compared to Ad-GFP control (*P* < 0.05 or 0.01; Figure 7(b)). The overexpression of Rap1GAP led to an increase in the ratio of cleaved-caspase-3/caspase-3 (*P* < 0.05; Figure 6(d)). As shown in Figure 7(c) and Figure S2, the apoptotic rate of NRCMs infected with Ad-Rap1GAP was significantly increased compared to that of Ad-GFP control (*P* < 0.01 or *P* < 0.05).

### 3.6. The Effects of Rap1GAP Overexpression/Knockout on Oxidative Stress Are Reversed by Autophagy Inducer/Inhibitor

**Figure 8:**
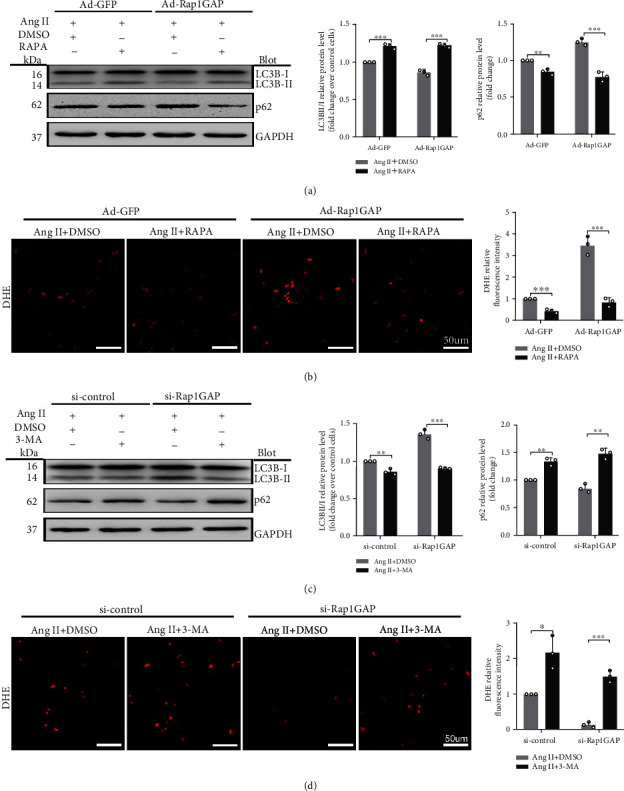
Reduced autophagy induced by Rap1GAP is associated with ROS production. (a) Western blot analysis for the expression of LC3B and p62 in Rap1GAP overexpressing cardiomyocytes treated with rapamycin. (b) ROS production in Rap1GAP overexpressing cardiomyocytes after intervention with rapamycin, determined using DHE fluorescent dye staining. Scale bar: 50 *μ*m. (c) Western blot analysis for LC3B and p62 in Rap1GAP knockdown cardiomyocytes treated with 3-Methyladenine. (d) ROS production in Rap1GAP knockdown cardiomyocytes treated with 3-Methyladenine, determined using DHE fluorescent dye staining. Scale bar: 50 *μ*m. ^∗^*P* < 0.05 and ^∗∗^*P* < 0.01. Results are the mean ± SEM of three independent experiments (*n* = 3).

To confirm the relationship between autophagy and oxidative stress, we treated NRCMs with autophagy inhibitor 3-Methyladenine (3-MA) and autophagy agonist rapamycin (RAPA) for 1 hour before the treatment with Ang II. Cells were harvested after exposure to Ang II for 24 hours. Compared with the Ad-Rap1GAP+Ang II group, the level of LC3BII/I was significantly increased and p62 was suppressed in RAPA-treated cardiomyocytes (*P* < 0.05 or *P* < 0.01; Figure 8(a)). Moreover, it attenuated the increase of ROS caused by Rap1GAP overexpression (*P* < 0.01; Figure 8(b)). In contrast, compared with the si-Rap1GAP+Ang II group, the content of LC3II/I was decreased and p62 was increased in 3-MA-treated NRCMs (*P* < 0.01; Figure 8(c)). Similarly, the ROS reduction caused by Rap1GAP knockdown was increased in the 3-MA-treated group (*P* < 0.05 or *P* < 0.01; Figure 8(d)).

## 4. Discussion

To our knowledge, this is the first study demonstrating that Rap1GAP is a critical mediator in Ang II-induced cardiomyocyte hypertrophy. Rap1GAP is a member of the Ras superfamily. The Ras family has been intensively studied in the field of cancer research, but rarely in cardiovascular diseases. It has been reported that the knockout of H-Ras, another member of the Ras subfamily, can prevent Ang II-induced arterial hypertension and ventricular remodeling [19, 20]. Ramos-Kuri et al. found that the Ras mutant Ras-Val12 or the dominant negative mutation N17-DN-Ras induced cardiac hypertrophy and produced cardiotoxicity [21]. As a member of RAS superfamily, Rap1GAP is an important tumor suppressor in tumor tissues, which inhibits cell proliferation, migration, and angiogenesis by attenuating the level of adhesion proteins that regulate cancer cell invasion, thereby increasing apoptosis and exerting tumor suppressive effects [22, 23]. In the present study, we found that Rap1GAP was expressed in cardiomyocytes and elevated in Ang II-induced hypertrophic cardiomyocytes. Moreover, Rap1GAP was also increased in the heart of Ang II-treated rats compared to control rats. Knockdown of Rap1GAP attenuated while overexpression of Rap1GAP accelerated Ang II-induced oxidative stress, apoptosis, and hypertrophy in cardiomyocytes. Meanwhile, Rap1GAP was closely related to cell autophagy. These results demonstrate a new role of Rap1GAP in the heart, which is the involvement of cardiac hypertrophy and remodeling.

The hypoxic environment is caused by increased cardiac oxygen consumption during the compensatory stage of cardiac hypertrophy. However, the disorders of oxygen metabolism eventually lead to heart failure. We hypothesized that Rap1GAP mediates the conversion of Rap1 from an active form to an inactive form, resulting in increased hypoxia in cardiomyocytes, and the increased ROS production aggravates myocardial damage. Yang et al. showed that activated Rap1 acts as a negative regulator of mitochondrial ROS production in the heart. The active form of Rap1 (Rap1GTP) was reduced by selective inhibition of Epac2 in adult rat ventricular myocytes [13]. Studies on other cell types showed that Rap1GAP increases ROS production by activating NADPH oxidase in retinal pigment epithelial cells and reduces choroidal neovascularization [24]. Our data in cardiomyocytes are consistent with their results, demonstrating that ROS was significantly increased by Rap1GAP upregulation in hypertrophic cardiomyocytes, while it was inhibited by Rap1GAP knockdown. Our work showed that Rap1GAP mediates cardiac remodeling by increasing ROS production.

Recent studies showed that autophagy plays a crucial role in Ang II-induced cardiac hypertrophy. Our data indicate that Rap1GAP inhibits autophagy in cardiomyocytes, resulting in autophagosome formation and mitochondrial damage. Uncontrolled autophagic disorders and mitochondrial disruption block the energy metabolism of cardiomyocytes and ultimately lead to cardiomyocyte death [25, 26]. Studies showed that autophagy also reduces oxidative stress damage by phagocytosis and degradation of oxidative derivatives [10, 27]. Here, we demonstrate the relationship between Rap1GAP-mediated autophagy and oxidative stress by using the inhibitors and inducers of autophagy. 3-Methyladenine exerts an effect of suppressing autophagy by inhibiting the autophagosome formation, while rapamycin is a macrolide immunosuppressant that specifically inhibits mTOR activation and activates autophagy by reducing phosphorylated mTOR. We have shown that oxidative stress was increased by blocking increased autophagy associated with Rap1GAP knockdown. In contrast, Rap1GAP overexpression-induced increase of ROS was attenuated by the activation of autophagy.

Apoptosis plays an important role in the pathological process of cardiac hypertrophy to heart failure. The increased cardiomyocyte apoptosis has been reported in Ang II-induced cardiac hypertrophy. In the compensatory stage of cardiac hypertrophy, apoptosis causes a progressive decrease in the cardiac functional contractile unit, while viable cardiomyocytes are adaptive hypertrophic accompanied by myocardial fibrosis. Numerous studies have found that both autophagy and oxidative stress induce apoptosis. Here, we demonstrate that Rap1GAP induces cardiomyocyte apoptosis which might be a main mechanism of Rap1GAP in cardiovascular diseases.

Numerous signaling molecules are involved in the autophagy regulation. AMP-activated protein kinase (AMPK) is a positive regulator of autophagy and plays a key role in maintaining energy balance. However, the abnormal AMPK also participates in the pathogenesis of cardiac hypertrophy, inflammatory response, and myocardial fibrosis by affecting cellular metabolisms. Mitochondrial ROS is a physiological activator of AMPK, and AMPK can alleviate the impaired redox balance caused by reactive oxygen stress by enhancing the bioavailability of oxides [28]. Findings from the present study demonstrated that Rap1GAP functions in cardiomyocytes through its regulation on AMPK and its downstream targets. AKT regulates cell growth and survival, and it exerts antiapoptotic effects by phosphorylating target proteins through various downstream pathways. mTOR is a downstream effector of AKT that mediates cellular nutrient metabolism and aging [29]. The AMPK/AKT/mTOR signaling pathway is a classical autophagy pathway in various tissues such as the heart, liver, and endothelium [30, 31]. This study has demonstrated that Rap1GAP increases the phosphorylation of AKT and mTOR by regulating AMPK phosphorylation, reduces the phosphorylation level of p70s6k, which is a target downstream of mTOR, and finally inhibits autophagy in cardiomyocytes.

**Figure 9:**
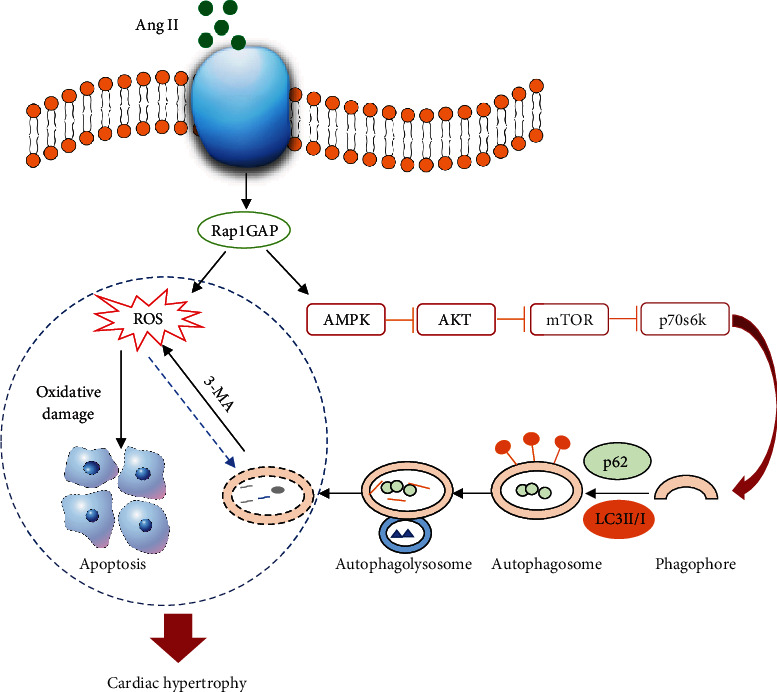
Hypothesized mechanisms of the role of Rap1GAP in Ang II-induced cardiomyocyte hypertrophy. Rap1GAP inhibits autophagy by suppressing the AMPK/AKT/mTOR signaling pathway and increases ROS production in Ang II-induced hypertrophic cardiomyocytes. Inhibition of autophagy reduces ROS clearance and further aggravates cardiac injury.

In summary, this study demonstrates that Rap1GAP is a new mediator of Ang II-induced cardiomyocyte hypertrophy through its regulating on cardiac autophagy, oxidative stress, and apoptosis by mediating the AMPK/AKT/mTOR signaling pathway. It might be a potential therapeutic target for the treatment of cardiac hypertrophy and heart failure, which is worth further investigations. However, we also realized the limitations of this study. Although the present data show the involvement of Rap1GAP in Ang II-induced cardiomyocyte hypertrophy, more specific animal models including cardiomyocyte-specific Rap1GAP knockout mouse are needed to further determine the critical roles of this new player in the pathogenesis of cardiac hypertrophy and other cardiovascular diseases. Based on our findings, we hypothesized the potential mechanisms of the role of Rap1GAP in Ang II-induced cardiomyocyte hypertrophy, as shown in Figure 9. However, our ongoing experiment of mass spectrometry analysis might be able to reveal more significant signaling pathways that are involved in the functional roles of Rap1GAP in cardiovascular diseases. Since Rap1GAP is a newly discovered protein and this report is the first showing its functions in the cardiomyocytes, there are still a lot of unknowns about this protein in the heart and several projects are under planning in our lab to further investigate the regulation and cardiac functions of this new protein.

## Data Availability

All data sets generated/analyzed for this study are included in the manuscript and the Supplementary Files.

## Supplementary Materials

Supplementary Materials There are 2 figures in the supplementary material. Figure S1: results of the gene transduction rate of Rap1GAP viral vector. Figures S2: analysis of the effect of Rap1GAP overexpression on cardiomyocyte apoptosis by flow cytometry.
