# Supplementary material for: Rap1GAP Mediates Angiotensin II-Induced Cardiomyocyte Hypertrophy by Inhibiting Autophagy and Increasing Oxidative Stress
Source: Oxid Med Cell Longev. 2021 Apr 15;2021:7848027. doi: 10.1155/2021/7848027 (PMC8062190; doi:10.1155/2021/7848027)
Supplement: Supplementary Materials — There are 2 figures in the supplementary material. Figure S1: results of the gene transduction rate of Rap1GAP viral vector. Figures S2: analysis of the effect of Rap1GAP overexpression on cardiomyocyte apoptosis by flow cytometry. [file 7848027.f1.docx]

**Figure S1.** (A) Representative images from fluorescence microscope showing fluorescence of GFP. (B) Flow cytometric analysis for detecting fluoresence protein GFP.
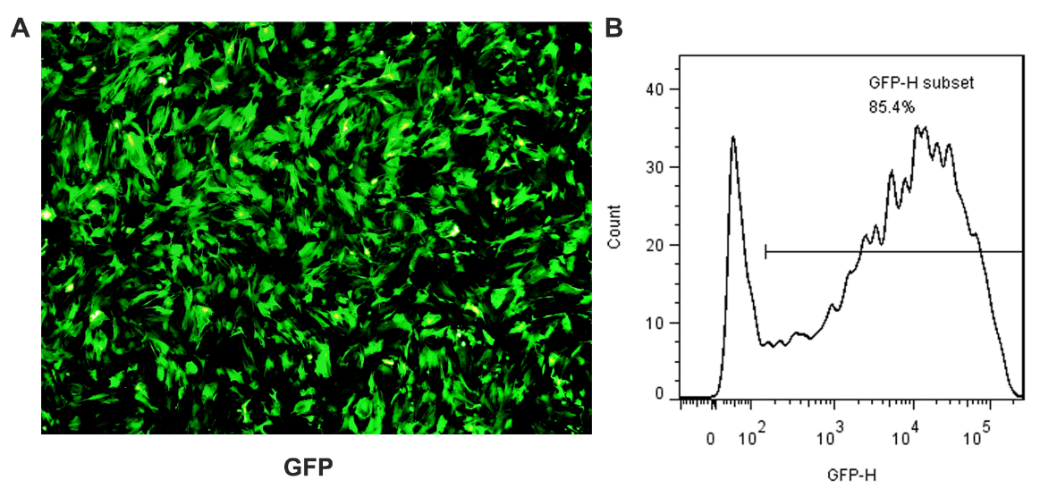


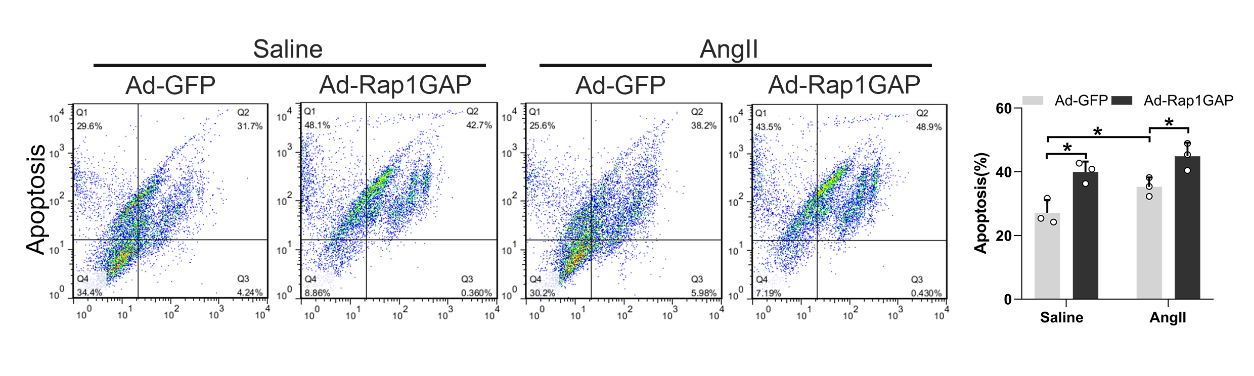
 **Figure S2.** (A) Flow cytometric analysis for NRCMs apoptosis stained with 7AAD and Annexin V-PE. *P < 0.05 and ** P < 0.01. Datas are the mean ± SEM of three independent experiments.
